# Identifying and Intervening in Child Maltreatment and Implementing Related National Guidelines by Public Health Nurses in Finland and Japan

**DOI:** 10.1155/2017/5936781

**Published:** 2017-02-06

**Authors:** Kayoko Suzuki, Eija Paavilainen, Mika Helminen, Aune Flinck, Natsuko Hiroyama, Taiko Hirose, Noriko Okubo, Motoko Okamitsu

**Affiliations:** ^1^School of Nursing, Tokyo Ariake University of Medical and Health Sciences, Tokyo, Japan; ^2^School of Health Sciences, University of Tampere, Etelä-Pohjanmaa Hospital District, Tampere, Finland; ^3^School of Health Sciences, University of Tampere and Science Center, Pirkanmaa Hospital District, Tampere, Finland; ^4^School of Health Sciences, University of Tampere, National Institute for Health and Welfare, Department of Government Services, Tampere, Finland; ^5^Graduate School of Health Care Sciences, Tokyo Medical and Dental University, Tokyo, Japan

## Abstract

*Aim. *This study aimed to investigate how public health nurses identify, intervene in, and implement the guidelines on child maltreatment in Finland and Japan and to compare the data between the two countries.* Method. *This study employed a cross-sectional design. Public health nurses' knowledge and skills with respect to child maltreatment prevention were assessed using a questionnaire consisting of three categories: identification, intervention, and implementation of guidelines. Public health nurses working in the area of maternal and child health care in Finland (*n* = 193) and Japan (*n* = 440) were the participants.* Results*. A significantly higher percentage of Japanese public health nurses identified child maltreatment compared to Finnish public health nurses, while Finnish nurses intervened in child maltreatment better than their Japanese counterparts. In both countries, public health nurses who had read and used the guidelines dealt with child maltreatment better than those who did not.* Conclusion. *The results suggest that effective training on child maltreatment and the use of guidelines are important to increase public health nurses' knowledge and skills for identifying and intervening in child maltreatment.

## 1. Introduction

The situation with respect to child well-being has rapidly improved in most developed countries including Finland and Japan during the last few decades. Finland and Japan are top ranked countries in the international comparison survey on child well-being in aspects such as health, safety, education, and environment [1]. Awareness of child maltreatment and its consequences on the future well-being and development of children has also increased during last decades. Considerable resources have been deployed to tackle the problem but not always to the maximum effect [2]. The number of child maltreatment cases has continued to increase in Japan. In 2013, 73,802 cases were reported to the Child Protection Service in Japan. The number was 2.8 times as many as that reported in 2003 (26,569) [3]. The context is similar in Finland, where the number of children in emergency placement has been growing over the last decade. A total of 4,192 of children were taken into emergency placement in 2013 in Finland. The number increased 3.3 times in 10 years as compared to that reported in 2003 (1,267) [4]. Child maltreatment is therefore a social issue in both countries. Parents of maltreated children are often known to a range of professionals, and it is crucial for professionals to be alert to signals of maltreatment, because children who are abused are unlikely to express the need for or seek help directly from statutory agencies; furthermore, their parents are likely to be ill-equipped to seek and make use of informal support services [5].

Childhood maltreatment is associated with a variety of changes in brain structure and function and in stress-responsive neurobiological systems [6–8]. In addition, adverse experiences in early childhood can have long-term health consequences, both physical and psychological [8]. Consequences of child maltreatment include impaired lifelong physical and mental health such as posttraumatic stress disorder or depression, and the social and occupational outcomes can ultimately slow the economic and social development of the country [9, 10]. Therefore, early childhood is a critical time for the promotion of healthy development and the prevention of mental disorders in adult life.

There are public health care systems in place to support children and their families in Finland and Japan. Public health nurses (PHNs) working with children and their families provide preventive health care services and play an important role as front workers in identifying, intervening in, and preventing child maltreatment. It is important for nurses to detect signs of children and families being at a risk of maltreatment. Preventive intervention in child maltreatment is practiced based on guidelines in both countries. In Finland, the National Nursing Guideline for identifying and intervening in child maltreatment based on systematic review was published by the Nursing Research Foundation in 2008. The main purpose of the guideline was to find methods with which it is possible to identify child maltreatment cases and, following that, continue to help children and families by finding ways out of their situation [11, 12]. In Japan, the guideline for child maltreatment prevention was designed by Ministry of Health, Labour and Welfare in 1999 and revised several times [13]. The contents of the guideline are mainly the definition of child maltreatment, prevention, and intervention in child maltreatment and child protection. The national guideline was arranged by each health care center and utilized. However, identifying and intervening in child maltreatment is extremely difficult and complex. Child maltreatment is mostly identified when children already have serious consequences and the sensitivities and specificity of tools are inadequate. Failure to consider the possibility of maltreatment will mean that appropriate diagnosis is not made and the child is returned to an abusive environment [14]. Therefore, research to improve the assessment of child maltreatment is needed for preventive nursing intervention.

The aims of this study were to investigate how PHNs identify, intervene in, and implement guidelines on child maltreatment in Finland and Japan and to compare the results between the two countries. The findings will be helpful to increase PHNs' knowledge and skills to prevent child maltreatment.

## 2. Methods

### 2.1. Design and Participants

This study employed a cross-sectional design to investigate how PHNs identify, intervene in, and implement guidelines on child maltreatment in Finland and Japan. The knowledge and skills of PHNs with respect to child maltreatment were assessed using a self-assessment questionnaire. Participants in this study were PHNs working in the area of maternal and child health care in both countries.

*Finland*. Participants were recruited from the register of the Finnish Union of Public Health Nurses. An electronic questionnaire was sent to PHNs who had an e-mail address and worked in child-related clinics (*n* = 800) in February 2012, among whom 367 nurses completed the questionnaire, resulting in a response rate of 46%. The survey was approved by the board of the Public Health Nurse Union. A letter stating the aim and details of this study was attached to the questionnaire and emphasized that the respondents would remain anonymous. Replying to the electronic questionnaire indicated informed consent.

*Japan*. The survey was conducted from September to October in 2012. The Japanese public health care system was classified into 5 types according to the population size of the municipality. A total of 700 health care centers were selected using a stratified sampling method according to the number of PHNs working in 5 types of health care centers in 2011. Similarly, 486 health care centers in small municipalities were selected by stratified sampling based on the number of PHNs working in the municipalities of the 47 prefectures. Research randomizer was used for the stratified sampling. Two sets of questionnaires were sent by mail to the PHNs who worked with pregnant women, children, and their families in a total of 700 health care centers (*n* = 1,400), of which 453 nurses completed the questionnaire (response rate: 32.4%). A cover letter was attached to the questionnaire to explain the aim and details of the study. It was emphasized that participation in the study was voluntary and participants would remain anonymous. Replying to the questionnaire indicated informed consent. This study was conducted with the approval of the Ethics Committee of Tokyo Medical and Dental University.

### 2.2. Measures

A questionnaire to assess PHNs' knowledge and skills with respect to child maltreatment was developed based on the research of Paavilainen et al. [15] based on the Finnish National Guideline. The context of the Finnish Guideline was suitable also for the Japanese context. The questionnaire consisted of 3 categories: identification (8 statements), intervention (31 statements), and implementation of guidelines (8 statements) on child maltreatment. Participants were asked to rate, on a 6-point Likert scale ranging from 1 (totally disagree) to 6 (totally agree), the extent to which they agreed with the statements. The questionnaire included demographic questions (age, gender, work experience as a PHN, and current job) and questions concerning whether PHNs knew about the existence of guidelines on child maltreatment and had read them. Before conducting this survey, 10 Finnish PHNs participated in a pilot test of the electronic version of the questionnaire, and subsequently a part was modified.

In Japan, the same questionnaire translated into Japanese was used in the survey. Japanese participants were asked to rate their agreement with the statements using a 4-point Likert scale ranging from 1 (totally disagree) to 4 (totally agree). A questionnaire using 4-point Likert scale is widely used in Japan. A scale which had wide range of choices was difficult to answer and unsuitable for Japanese participants. Therefore 4-point Likert scale was adapted for the Japanese questionnaire instead of 6-point scale. One item about intervention in child maltreatment was excluded from the Japanese questionnaire because it was difficult to answer in Japanese.

### 2.3. Data Analysis

To make the groups as comparable as possible, data from PHNs working in maternity, family planning, or child clinics in Finland (*n* = 193) and data from Japanese participants who worked with pregnant women, children, and their families (*n* = 440) were used for the analysis.

The answers for each item on the questionnaire were classed as either “disagree” (answers of 1–3 points in Finland and 1-2 points in Japan) or “agree” (answers of 4–6 points in Finland and 3-4 point in Japan). Using chi-square tests, we examined whether there were significant differences between Finnish and Japanese participants. Statistical analyses were conducted using SPSS.

## 3. Results

### 3.1. Demographic Characteristics

The demographic characteristics of the participants are shown in Table 1. Most participants were female in Finland and Japan. Approximately 80% of Finnish participants knew about the guidelines; however, only 53.9% of them had read them. On the other hand, about 95% of Japanese participants knew about the guidelines and 88% of them had read them. There were significant differences between these answers from Finnish and Japanese PHNs (*P* < 0.001).

### 3.2. Identification of Child Maltreatment

On all of the items regarding the identification of child maltreatment, the percentage of Japanese PHNs with “agree” answers was significantly higher than Finnish PHNs (Table 2). More than 90% of Japanese participants responded that they recognized risk factors of child maltreatment related to children, parents, and families. Finnish respondents agreed that they recognized child maltreatment based on parents-related risk factors and family-related risk factors moderately well, although more than 60% of respondents provided “disagree” answers on three items; PHNs recognized child maltreatment based on the child's behavior and the parents' behavior, and they recognized mental signs sufficiently well.

### 3.3. Intervention in Child Maltreatment

Concerning the intervention to support children and their families, the percentage of Finnish participants who responded with “agree” was higher than Japanese participants for all items. There were significant differences between answers of Finnish and Japanese PHNs in all the items except one item regarding support when the child has special needs or illness (Table 3).

For most items about intervention when suspecting child maltreatment, the rate of participants who responded with “agree” was significantly higher for Finland than Japan. More than 90% of Finnish PHNs responded that they always made a child welfare notification, documented the maltreated child, guided to follow-up treatment, and listened to the family under suspicion. More than 90% of Japanese PHNs responded with “agree” for an item about documentation. The percentage of PHNs reporting that they helped the maltreated child and the family sufficiently well was less than 40% in Japan (Table 4).

Regarding the items on intervention, collaboration, and support from other professionals, the percentage of Finnish PHNs who responded with “agree” was significantly higher than Japanese PHNs in 5 items: collaboration with other professionals, support from peers, support from physicians, clear instructions on how to make a child welfare notification, and working according to the child maltreatment guidelines. The rate of Finnish PHNs who responded with “agree” was significantly lower than Japanese PHNs for 2 items about support from superiors and instructions to handle child maltreatment cases. Over 90% of PHNs in both countries reported that they knew who to contact when suspecting child maltreatment, and there were no significant differences between the two countries for this item (Table 5). In addition, more than 80% of PHNs in Finland and Japan reported getting enough support from peers when child maltreatment was suspected, and the rates were the highest for support from other professionals in both countries. Although 78% of Finnish PHNs reported receiving enough support from physicians, this rate was only 29% for Japanese PHNs.

### 3.4. Implementation of Guidelines

Answers regarding the implementation of guidelines are shown in Table 6. The percentage of Finnish participants who responded with “agree” was significantly higher than Japanese participants for all items regarding the implementation of guidelines. Although more than 40% of Finnish PHNs had not read the guidelines, 90% of them reported that the guidelines were an important tool. Although 90% of PHNs had read the guidelines in Japan, only 55% of them recognized them as an important tool. The percentage of Japanese respondents who reported having familiarized themselves with the contents of the guidelines (18%) was significantly lower than Finnish respondents (50%).

Data from the PHNs in each country were divided into two groups according to the answer given to the item on having familiarized themselves with the contents of the guidelines. PHNs' answers to the 8 items in the identification section and the 31 items in the intervention section were analyzed using a chi-square test to explore the differences between the answers from PHNs in the two groups. The results of the comparison, of the Japanese respondents who were familiar with the contents of the guideline with those who were not, showed significant differences in the responses to 7 of the identification items and 27 of the intervention items (the former group had a higher percentage of PHNs who responded with “agree”). Likewise, significant differences were observed in the Finnish respondents' answers to 4 of the identification items and 12 of the intervention items, when comparing those who were familiar with the contents of the guideline with those who were not. Thus, the percentage of participants who responded with “agree” was significantly higher in the group of PHNs who were familiar with the contents of the guideline, compared with the group of PHNs who were not.

## 4. Discussion

### 4.1. Identification and Intervention in Child Maltreatment

The current survey was conducted to investigate PHNs' knowledge and skills regarding child maltreatment prevention and data from Finnish and Japanese PHNs were compared. The results indicate that Japanese PHNs identify child maltreatment better than their Finnish counterparts, while Finnish PHNs intervene in child maltreatment better than Japanese PHNs. This may be due to the differences in the public health care systems of the two countries. In Finland, as a Nordic country, child health care services are provided for all children and their families, who can access these services 16 times before entering school. The services are free of charge and their rate of use is extremely high [16]. Therefore, PHNs in Finland can support all children and their families based on their needs. In Japan there is a public health care system, but the frequency of use of the universal child health care services is extremely lower than in Finland. It is therefore an important role of Japanese PHNs to identify maltreated children and to connect them with additional health care and welfare services, as the opportunities for PHNs to meet children and their families are limited [17]. It seems that this system might promote Japanese PHNs skills to identify child maltreatment.

Japanese PHNs evaluated their knowledge and skills for identifying child maltreatment more positively than for intervening in child maltreatment. This result is similar to that of an earlier study conducted on pediatric hospital staff [18]. The knowledge and skills of Finnish PHNs to intervene in child maltreatment were higher than those reported in that research [18]. It has already been reported that Finnish PHNs who had participated in training on child maltreatment intervened well in child maltreatment [15]. In Finland, PHNs' training was promoted in the context of the Early European Promotion Project (EEPP). The EEPP was conducted in five European countries including Finland during the first half of the 2000s, with the aim of promoting parent-infant relationships and hence infant mental health and of preventing emotional and behavioral problems by providing a universal service available to all families with small children. Training in the EEPP was intended to promote PHNs preventive work for young children and their families [19–21]. It seems that this training might have increased Finnish PHNs' knowledge and skills to intervene in child maltreatment. Therefore, it is suggested that effective PHNs' training, such as the EEPP training, is also needed for Japanese PHNs to increase their knowledge and skills regarding child maltreatment intervention.

### 4.2. Implementation of Guidelines on Child Maltreatment

Although most Japanese PHNs had read the guidelines on child maltreatment, most of them did not agree with the questionnaire statements regarding the implementation of the guidelines. This finding shows that, regardless of the guidelines, there are some issues in the process of their introduction and implementation. In addition, the Japanese PHNs who were familiar with the contents of the guidelines identified and intervened in child maltreatment better than the PHNs who did not. These results suggest that developing strategies for utilizing the guidelines are required in order to increase PHNs' knowledge and skills for identifying and intervening in child maltreatment in Japan.

In this study, Finnish PHNs who familiarized themselves with the contents of the guidelines dealt with child maltreatment better than PHNs who did not. The importance of guidelines has been shown in earlier research regarding child maltreatment [22]. Therefore, it is suggested that understanding the contents of guidelines is also important in order to increase the knowledge and skills for identifying and intervening in child maltreatment in Finland as well as in Japan.

### 4.3. Limitations of This Study

The knowledge and skills of PHNs to identify and intervene in child maltreatment were assessed using a self-report questionnaire in this study. It is considered that evaluating PHNs' knowledge and skills accurately to prevent child maltreatment in this way is difficult and this is a limitation of this study.

The survey was conducted using questionnaires, in the form of a web-based survey in Finland and a mail survey in Japan. The difference in the survey methods may have contributed to the differences in the answers of the respondents between the two countries.

Data from Finnish and Japanese PNHs were compared and discussed in this study. However, the factors associated with effective nursing interventions were not considered in detail. Further discussion about these factors is needed in order to promote PHNs' skills with respect to child maltreatment intervention.

## 5. Conclusion

The current study found that Japanese PHNs identified child maltreatment better than Finnish PHNs, and Finnish PHNs intervened in child maltreatment better than their Japanese counterparts. It was shown that effective training on child maltreatment is needed to increase PHNs' knowledge and skills for identifying and intervening in child maltreatment. In addition, utilizing guidelines is also required in order to effectively intervene in child maltreatment in Finland and Japan.

## Figures and Tables

**Table 1:** Background of the participants.

Variables	Finland (*n* = 193)		Japan (*n* = 440)
	Mean (SD)		
Age	42.1 (11.2)		37.3 (9.1)
Working years as a nurse	12.5 (10.3)		12.1 (9.1)
	*n* (percentages)		
Gender (male/female)	1 (0.5%)/192 (99.5%)		5 (1%)/434 (99.0%)
PHNs knowing the existence of the guidelines	156 (80.8%)		417 (95.4%)
PHNs who had read the guidelines	104 (53.9%)		383 (88.0%)

**Table 2:** Identification of child maltreatment.

Variables	Finland (*n* = 193)		Japan (*n* = 440)		*χ* ^2^(1)	Φ
	Disagree	Agree	Disagree	Agree		
PHNs meet maltreated children often	164 (85%)	29 (15%)	290 (67%)	145 (33%)	22.37^*∗∗∗*^	.19
*PHNs recognize child maltreatment based on*						
Child-related risk factors	88 (46%)	105 (54%)	25 (6%)	413 (94%)	114.99^*∗∗∗*^	.48
Parents-related risk factors	58 (30%)	135 (70%)	21 (5%)	418 (95%)	78.26^*∗∗∗*^	.35
Family-related risk factors	62 (32%)	131 (68%)	28 (6%)	411 (94%)	72.77^*∗∗∗*^	.34
The child's behavior	117 (61%)	76 (39%)	140 (32%)	299 (68%)	45.87^*∗∗∗*^	.27
The parents' behavior	130 (67%)	63 (33%)	131 (30%)	308 (70%)	77.84^*∗∗∗*^	.35
PHNs recognize physical signs sufficiently well	88 (46%)	105 (54%)	73 (17%)	366 (83%)	59.25^*∗∗∗*^	.31
PHNs recognize mental signs sufficiently well	120 (62%)	73 (38%)	111 (25%)	327 (75%)	78.32^*∗∗∗*^	.35

Estimate significance: ^*∗∗∗*^*P* < 0.001.

**Table 3:** Intervention to support children and their families.

Variables	Finland (*n* = 193)		Japan (*n* = 440)		*χ* ^2^(1)	Φ
	Disagree	Agree	Disagree	Agree		
*PHNs discuss sufficiently well with families *						
Risk factors in families	54 (28%)	139 (72%)	203 (47%)	233 (53%)	19.13^*∗∗∗*^	−.17
The child's care	35 (18%)	158 (82%)	140 (32%)	297 (68%)	12.90^*∗∗∗*^	−.14
Problems in the couple's relationship	70 (36%)	123 (64%)	258 (59%)	179 (41%)	27.81^*∗∗∗*^	−.21
Problems in their daily life	17 (9%)	176 (91%)	132 (30%)	304 (70%)	34.10^*∗∗∗*^	−.23
The child's development	21 (11%)	172 (89%)	92 (21%)	345 (79%)	9.41^*∗∗*^	−.12
Problems in the child's development	22 (11%)	171 (89%)	93 (21%)	343 (79%)	8.83^*∗∗*^	−.12
*PHNs advise parents sufficiently well to*						
Seek help when needed	9 (5%)	184 (95%)	117 (27%)	319 (73%)	41.05^*∗∗∗*^	−.26
Deal well with the child's tantrums	22 (11%)	171 (89%)	112 (26%)	324 (74%)	16.29^*∗∗∗*^	−.16
Act well when the child does not meet parents' expectations	31 (16%)	162 (84%)	148 (34%)	289 (66%)	20.55^*∗∗∗*^	−.18
Act well when the child has special needs or illness	52 (27%)	141 (73%)	143 (33%)	293 (67%)	2.14^NS^	−.06
Act well when the child cries	26 (13%)	167 (87%)	112 (26%)	323 (74%)	11.75^*∗∗*^	−.14
Discuss their consistent nurture	35 (18%)	158 (82%)	135 (31%)	300 (69%)	11.27^*∗∗*^	−.13

Estimate significance: NS = not significant; ^*∗∗*^*P* < 0.01; ^*∗∗∗*^*P* < 0.001.

**Table 4:** Intervention when suspecting child maltreatment.

Variables	Finland (*n* = 193)		Japan (*n* = 440)		*χ* ^2^(1)	Φ
	Disagree	Agree	Disagree	Agree		
*When suspecting child maltreatment PHNs*						
Ask about it directly	48 (25%)	145 (75%)	247 (57%)	189 (57%)	54.26^*∗∗∗*^	−.29
Always make a child welfare notification	16 (8%)	177 (92%)	133 (31%)	303 (69%)	36.52^*∗∗∗*^	−.24
Help the maltreated child sufficiently well	86 (45%)	107 (55%)	265 (61%)	170 (39%)	14.51^*∗∗∗*^	−.15
Help the family sufficiently well	88 (46%)	105 (54%)	282 (65%)	154 (35%)	20.11^*∗∗∗*^	−.18
Document the maltreated child	17 (9%)	176 (91%)	41 ( 9%)	396 (91%)	0.05^NS^	−.01
Guide to follow-up treatment	17 (9%)	176 (91%)	176 (40%)	259 (60%)	62.91^*∗∗∗*^	−.32
Listen to the family under suspicion	14 (7%)	179 (93%)	96 (22%)	341 (78%)	20.11^*∗∗∗*^	−.18

Estimate significance: NS = not significant; ^*∗∗∗*^*P* < 0.001.

**Table 5:** Intervention, collaboration, and support from other professionals.

Variables	Finland (*n* = 193)		Japan (*n* = 440)		*χ* ^2^(1)	Φ
	Disagree	Agree	Disagree	Agree		
*When suspecting child maltreatment PHNs*						
Collaborate sufficiently well with other professionals	9 (5%)	184 (95%)	103 (24%)	331 (76%)	33.11^*∗∗∗*^	−.23
Think multi-professional collaboration works well in the municipality	73 (38%)	120 (62%)	151 (35%)	285 (65%)	0.59^NS^	.03
Think multi-professional collaboration works well in their organization	46 (24%)	147 (76%)	131 (30%)	305 (70%)	2.55^NS^	−.06
Know who to contact	17 (9%)	176 (91%)	22 (5%)	415 (95%)	3.28^NS^	.07
*When suspecting child maltreatment PHNs get enough support from*						
Superiors	85 (44%)	108 (56%)	92 (21%)	345 (79%)	35.02^*∗∗∗*^	.24
Peers	17 (9%)	176 (91%)	70 (16%)	366 (84%)	5.90^*∗*^	−.10
Physicians	43 (22%)	150 (78%)	307 (71%)	128 (29%)	126.39^*∗∗∗*^	−.45
Child protection	52 (27%)	141 (73%)	139 (32%)	298 (68%)	1.50^NS^	−.05
*In the office/clinic*						
PHNs have joint instructions to deal with child maltreatment	75 (39%)	118 (61%)	128 (29%)	309 (71%)	5.61^*∗*^	.09
PHNs have clear instructions how to make a child welfare notification	45 (23%)	148 (77%)	179 (41%)	257 (59%)	18.36^*∗∗∗*^	−.17
It is possible to work according to the guidelines	58 (30%)	135 (70%)	181 (42%)	250 (58%)	8.05^*∗∗*^	−.11

Estimate significance: NS = not significant; ^*∗*^*P* < 0.05; ^*∗∗*^*P* < 0.01; ^*∗∗∗*^*P* < 0.001.

**Table 6:** Implementation of guidelines on child maltreatment.

Variables	Finland (*n* = 193)		Japan (*n* = 440)		*χ* ^2^(1)	Φ
	Disagree	Agree	Disagree	Agree		
The guidelines are an important tool	20 (10%)	173 (90%)	196 (45%)	237 (55%)	71.96^*∗∗∗*^	−.34
The guidelines lead my work	65 (34%)	128 (66%)	223 (52%)	210 (48%)	17.07^*∗∗∗*^	−.17
The guidelines change my work routines to comply with the recommendations	13 (7%)	180 (93%)	372 (87%)	56 (13%)	362.97^*∗∗∗*^	−.77
PHNs have had enough training on the contents of the guidelines	147 (76%)	46 (24%)	376 (87%)	57 (13%)	11.06^*∗∗*^	−.13
PHNs have familiarized themselves with the contents of the guidelines	96 (50%)	97 (50%)	355 (82%)	78 (18%)	68.92^*∗∗∗*^	−.33
PHNs discuss the contents of the guidelines in the office	144 (75%)	49 (25%)	373 (86%)	60 (14%)	12.35^*∗∗∗*^	−.14
PHNs support each other to act in accordance with the guidelines in the office	90 (47%)	103 (53%)	321 (74%)	112 (26%)	44.78^*∗∗∗*^	−.27
There are sufficient resources to implement the guidelines in the office	110 (57%)	83 (43%)	394 (91%)	38 (9%)	100.00^*∗∗∗*^	−.40

Estimate significance:  ^*∗∗*^*P* < 0.01; ^*∗∗∗*^*P* < 0.001.
